# Cerebrospinal Fluid Total, Phosphorylated and Oligomeric A-Synuclein in Parkinson’s Disease: A Systematic Review, Meta-Analysis and Meta-Regression Study

**DOI:** 10.3390/biomedicines12102266

**Published:** 2024-10-05

**Authors:** Ioanna Kapsali, Maria-Evgenia Brinia, Vasilios C. Constantinides

**Affiliations:** 1 Neurodegenerative Disorders and Epilepsy Ward, First Department of Neurology, Eginition Hospital, National and Kapodistrian University of Athens, 11528 Athens, Greece; ioannakapsali@ymail.com (I.K.); mariaevgeniabr@gmail.com (M.-E.B.); 2 Neurochemistry and Biomarkers Unit, First Department of Neurology, Eginition Hospital, National and Kapodistrian University of Athens, 11528 Athens, Greece

**Keywords:** synuclein, Parkinson’s disease, cerebrospinal fluid, biomarkers, review, meta-analysis

## Abstract

**Background**: The diagnostic accuracy for Parkinson’s disease (PD), a synucleinopathy, based on diagnostic criteria is suboptimal. A biomarker for synucleinopathies is pivotal both from a clinical and from a research point of view. CSF a-synuclein has been extensively studied over the past two decades as a candidate biomarker of synucleinopathies. Herein, we present data on studies focusing on total, phosphorylated and oligomeric CSF a-synuclein in PD. **Methods**: Pubmed, Scopus and Web of Science were searched for studies with >10 PD patients and control subjects, with data (mean, SD) on total, phosphorylated or oligomeric a-synuclein. Cohen’s *d*, as a measure of effect size, was calculated for all a-synuclein forms. Subgroup analysis and meta-regression were performed in an effort to explain between-study heterogeneity. **Results**: Thirty studies on total, six studies on oligomeric and one study on phosphorylated a-synuclein were included. Total a-synuclein was decreased and oligomeric a-synuclein increased in PD patients vs. controls. The effect size was medium for total and high for oligomeric a-synuclein. A-syn forms provided suboptimal combined sensitivity/specificity for the differentiation of PD from controls. There was significant between-study heterogeneity. The PD cohort characteristics (sex, age, disease duration, UPDRS, H & Y) and study characteristics (study design, healthy vs. neurological controls, control for CSF blood contamination, method of a-syn measurement) could not account for between-study heterogeneity. Publication bias was limited. **Conclusions**: CSF a-synuclein levels lack sufficient accuracy to be used as biomarkers for PD. The standardization of (pre)analytical variables may improve the discriminatory power of a-synuclein forms in the future.

## 1. Introduction

Parkinson’s disease (PD) is the most common neurodegenerative movement disorder, affecting approximately 1% of persons over the age of 60 years [1]. PD was traditionally defined by the presence of asymmetrical rest tremor, bradykinesia and rigidity. Data over the past three decades, particularly from epidemiological and genetic studies, have shifted the definition of PD from a disorder manifesting exclusively with motor symptoms to a multi-system disorder, with early non-motor manifestations (such as depression, REM sleep behavior disorder (RBD), hyposmia and constipation), and the emergence of cognitive decline, gait difficulties and severe dysautonomia as the disease progresses [2].

Pivotal to our understanding of Parkinson’s disease was the discovery of Lewy bodies and Lewy neurites, which are characterized by aggregated, abnormally misfolded a-synuclein (a-syn), a mainly synaptic, 140-amino acid protein expressed by neurons [3]. This discovery has shifted the theoretical framework of Parkinson’s disease from a strictly clinical entity to a neuropathological entity, with distinct neuropathological lesions characterized by a specific proteinopathy.

This conceptual shift has highlighted the importance of introducing biomarkers with molecular specificity for PD (and synucleinopathies in general), in an effort to increase diagnostic accuracy on a clinical level. The implementation of biomarkers is also pivotal on a research level, since it would facilitate the accurate recruitment of PD patients in clinical studies of potential disease-modifying agents with molecular specificity, even in oligosymptomatic or preclinical stages of the disease [4].

Following the paradigm of Alzheimer’s disease, where cerebrospinal fluid biomarkers of amyloid pathology, tau pathology and neurodegeneration have already been discovered, multiple studies over the past two decades have focused on the potential of cerebrospinal fluid total a-syn as a candidate biomarker of synucleinopathies using diverse methodologies (e.g., enzyme-linked immunosorbent assay (ELISA), bead-based immunoassays such as Luminex, etc.) [5]. Over time, the focus has shifted to phosphorylated and oligomeric forms of a-syn, as post-translational alterations in a-syn seem to drive neurodegeneration.

Recent meta-analyses have highlighted the suboptimal diagnostic performance of CSF a-syn in PD [6,7], due in part to high heterogeneity among the included studies. To date, subgroup analysis and meta-regression have not been performed to examine the effect of study design and PD cohort characteristics on this heterogeneity.

The present systematic review aims to present data regarding studies of CSF total, oligomeric and phosphorylated a-syn in PD compared to control groups. Through subgroup analysis and meta-regression, the effect of multiple study characteristics (study design; presence/absence of control for CSF blood contamination; method of CSF total a-syn measurement; healthy vs. neurological control subjects) and PD cohort characteristics (male/female ratio, age, disease duration, UPDRS III, H & Y) on inter-study heterogeneity will be examined, in an effort to determine the possible reasons underlying the lack of clinically applicable, a-syn-based biomarkers for PD to date.

## 2. Materials and Methods

For the purposes of the present study, the Preferred Reporting Items for Systematic Reviews and Meta-Analyses (PRISMA) statement was implemented [8]. The study protocol was registered in the International Prospective Register for systematic reviews (PROSPERO; ID: CRD42024500414). Due to the design of this study (systematic review) and the use of previously published data, no institutional board review approval was obtained.

### 2.1. Literature Search Strategy

PubMed, Web of Science Core Collection and Scopus were searched from database inception to 31/1/2024 by three authors independently (I.K., M-E.B. and V.C.C.). Only studies with a consensus among reviewers on study eligibility were included. A subsequent manual search was performed on all included studies regarding the following: (a) the citations of the included studies; (b) relevant studies (from PubMed); (c) the references of the included studies; (d) studies included in previous relevant systematic reviews and meta-analyses. The corresponding authors of the papers were contacted in an effort to retrieve the full texts, in cases of full text unavailability or non-extractability.

The search strategy applied was (CSF OR cerebrospinal fluid) AND (synuclein OR biomarker) AND (Parkinson OR Parkinsonism OR parkinsonian OR extrapyramidal OR movement disorder) in the study title.

### 2.2. Eligibility Criteria and Study Selection

The inclusion criteria were as follows:

(a) Publication in the English language;
(b) Original, peer-reviewed research papers;
(c) Studies with ≥10 PD patients and ≥10 control subjects;
(d) Studies including data on at least one of the following: total CSF a-syn, phosphorylated CSF a-syn or oligomeric (total, non-phosphorylated) CSF a-syn;
(e) Studies with available, extractable or retrievable (through scatterplots) mean values and standard deviations (mean, SD) of CSF a-syn.

The exclusion criteria were as follows:

(a) Non-original studies (reviews, meta-analyses);
(b) Case series with <10 PD patients or <10 control subjects and case reports;
(c) Abstracts in conferences or congresses of other scientific meetings;
(d) Studies implementing seeding assays for CSF a-syn (PMCA: protein misfolding cyclic amplification; RTQuIC: real-time quaking-induced conversion);
(e) Studies with identical patient cohorts.

In cases of publications with complete or partial overlap of patient cohorts, an algorithm was applied to reach a decision regarding the eligibility of the studies [9]. This algorithm included an evaluation of authorship, study characteristics, sample characteristics, constructs’ and measures’ definitions, and study effects.

### 2.3. Data Extraction

Data extraction was performed by three authors independently (I.K., M-E.B. and V.C.C.). All data were cross-checked for discrepancies among databases, and raw data from the manuscripts were re-evaluated when necessary.

The following data were excluded from the studies: first author; year of publication; study title; study design (i.e., retrospective, cross-sectional, prospective, longitudinal, unspecified, other); period of recruitment; center of study; methodology of a-syn quantification (i.e., ELISA, Luminex, other); presence/absence of control for CSF blood contamination.

Additionally, for the PD group and the control group, the following data were extracted where available: male/female ratio; mean age in years; mean disease duration in months (applicable only in the patient groups); total CSF a-syn, phosphorylated CSF a-syn and oligomeric CSF a-syn (in pg/mL).

In cases of missing (not reported) data, a review was performed on the Supplementary files of relevant papers. Mean and SD values were extracted from scatterplots, boxplot, or error bar plots where available via PlotDigitizer application (https://plotdigitizer.com/app, accessed on 30 January 2024).

### 2.4. Summary Measures

Cohen’s *d*, as a measure of standardized mean difference (SMD), was calculated to quantify the effect size of total, phosphorylated and oligomeric CSF a-syn levels for the distinction of PD patients from control subjects. Cohen’s *d* was interpreted as very small (*d* ≈ 0.01), small (*d* ≈ 0.2), medium (*d* ≈ 0.5), large (*d* ≈ 0.8), very large (*d* ≈ 1.2), or huge (*d* ≈ 2.0), based on recommendations [10].

### 2.5. Quality Evaluation

Quality evaluation was performed by three authors independently (I.K., M.-E.B., V.C.C.) by implementing the QUADAS-2 tool [11]. It includes four key domains—patient selection, index test, reference standard and flow/timing—which are assessed in relation to bias and concerns regarding applicability. For the purposes of the present meta-analysis, the following signaling questions were excluded due to non-applicability: (a) “Was a case–control design avoided?” from the patient selection domain; (b) “If a threshold was used, was it prespecified?” from the index test domain; (c) “Was there an appropriate interval between index tests and reference standard?” from the flow and timing domain. The signaling question “Was CSF a-syn measurement controlled for CSF blood contamination?” was added to the index test domain, as CSF blood contamination may result in inaccurate a-syn measurements.

Even when data regarding the signaling questions “Were the index test results interpreted without knowledge of the results of the reference standard” and “Were the reference standard results interpreted without knowledge of the results of the index test” were missing or not specified, this was not considered to significantly affect the applicability or increase the risk of bias of a study, considering that PD diagnosis is clinical and a-syn CSF measurement is automated and cannot be manipulated.

### 2.6. Statistical Analysis

The qualitative assessment of the presence or absence of heterogeneity was performed using the *Q* statistic, and the between-study heterogeneity was quantified by the *I*^2^ statistic. Heterogeneity was classified as low, moderate or high, with *I*^2^ values of <25%, 25–50% and >50%, respectively. A randomeffects model was applied for meta-analysis, in an effort to control for between-study heterogeneity. Cohen’s *d* was applied as a measure of the effect size of distinction between a-syn CSF levels in PD patients vs. the control group. Forest plots were produced displaying the effect sizes, standard errors, confidence interval limits, *p*-values and weights.

Funnel plots (with Cohen’s *d* on the x-axis and the standard error on the y-axis) were constructed in order to visualize any outlying studies, in an effort to test for publication bias. In order to quantify bias, an Egger linear regression test was performed.

### 2.7. Subgroup Analysis

Subgroup analysis was conducted to explore the effect of categorical variables on between-study variability. These included the following: (a) the study design (i.e., prospective vs. retrospective vs. cross-sectional vs. longitudinal); (b) the use of healthy controls vs. neurological control subjects; (c) the methodology applied for a-syn measurement (ELISA vs. Luminex); (d) the presence or absence of a control for CSF blood contamination. These analyses were performed exclusively for total CSF a-syn, where 30 studies were available. Due to a limited number of studies with data on oligomeric a-syn (n = 6) and phosphorylated a-syn (n = 1), subgroup analysis was not performed for these a-syn forms.

### 2.8. Meta-Regression

Univariate random-effects meta-regression models were employed to explore the source of between-study heterogeneity. The continuous variables (possible moderators) of PD cohorts included in these analyses were as follows: (a) male/female ratio; (b) mean age; (c) mean disease duration; (d) mean UPRDS III score; (e) mean H & Y score. Due to the limited number of studies with data on oligomeric a-syn (n = 6) and phosphorylated a-syn (n = 1), meta-regression was not performed for these a-syn forms. Bubble plots were produced to illustrate the association between possible moderators and effect size.

SPSS vs. 29 (IBM Corp. Released 2023; IBM SPSS Statistics for Windows, Version 29.0. Armonk, NY, USA: IBM Corp.) was used by one author (V.C.C.) for all statistical analyses. A two-tailed *p* value < 0.05 was considered statistically significant.

## 3. Results

### 3.1. Literature Search and Screening Results

**Figure 1:**
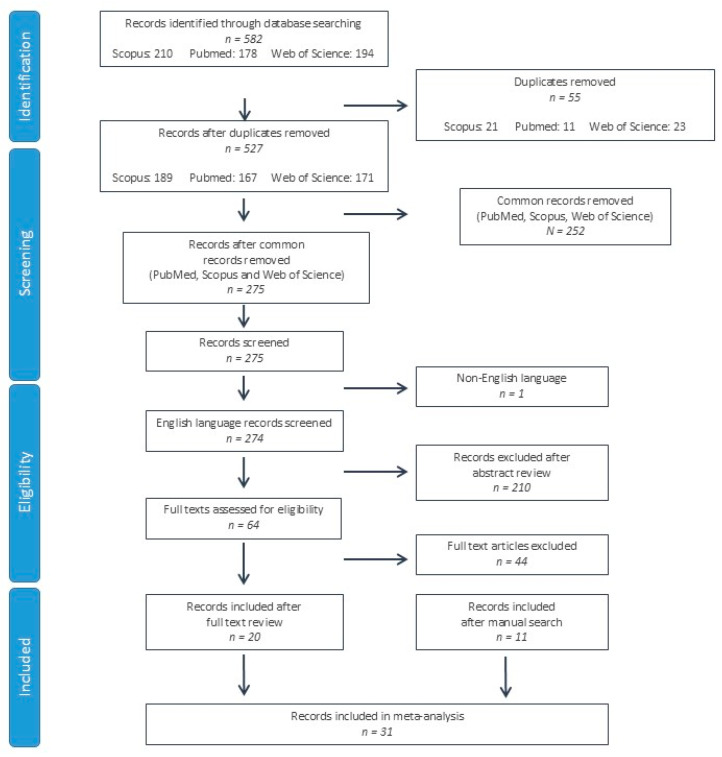
Flow chart of study selection according to PRISMA (Preferred Reporting Items for Systematic Reviews and Meta-Analyses) criteria.

A total of 582 studies were identified from the PubMed, Scopus and Web of Science databases. After duplicate record elimination, 275 records were screened by reviewing the title and abstract, eliminating 211 further studies. On the 64 remaining records, the full texts were reviewed, eliminating 44 records. In addition to the 20 records included after the full text review [12,13,14,15,16,17,18,19,20,21,22,23,24,25,26,27,28,29,30,31], 11 further studies were identified after a manual search of the references, citations and related articles of these records [32,33,34,35,36,37,38,39,40,41,42]. A total of 31 studies were included in the systematic review and meta-analysis (Figure 1).

### 3.2. Basic Features Included in the Study

The basic characteristics of the included studies are summarized in Table 1. Thirty studies included data on total CSF a-syn, a single study (with two cohorts) included data on phosphorylated CSF a-syn and six studies had data on oligomeric CSF a-syn. Regarding study design, thirteen cohorts (from twelve studies) were cross-sectional, eight cohorts (from seven studies) were prospective, two cohorts were longitudinal, one cohort was retrospective and thirteen cohorts had undefined study designs.

Twenty-four of the studies used ELISA for a-syn measurement, four studies implemented Luminex and the remaining studies used other methods. Ten studies (eleven cohorts) did not control for CSF blood contamination prior to a-syn quantification.

Six studies (with seven cohorts) had missing data on the male-to-female ratio in PD cohorts, and seven studies in control cohorts. Five studies (including seven cohorts) had missing data on the mean age of PD cohorts and four studies (six cohorts) on control subjects. Ten studies (with eleven cohorts) did not include data on the mean disease duration of PD patients (Table 1).

### 3.3. Quality Evaluation of Included Studies

**Figure 2:**
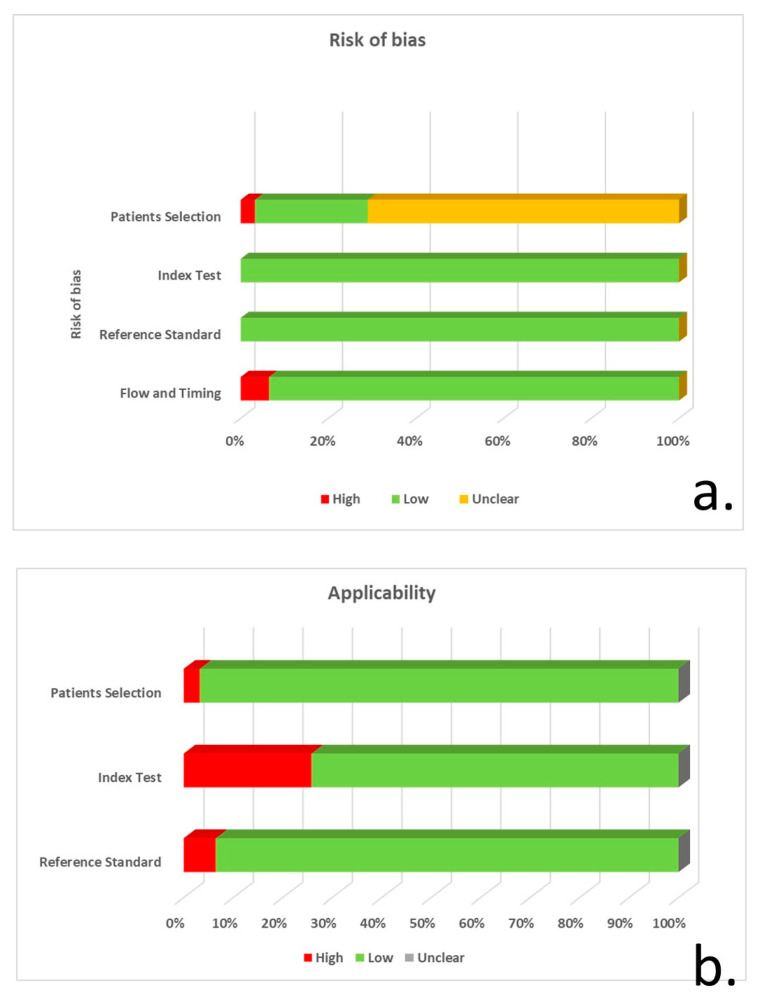
QUADAS-2 tool application for risk of bias (**a**) and concerns regarding the applicability of studies (**b**) for patient selection, index test, reference standard and flow/timing.

Based on the QUADAS-2 tool, 22 of the studies did not explicitly specify consecutive/random selection of patients, resulting in unclear risk of bias regarding patient selection. Eight studies had a low risk of bias, and a single study had a high risk due to unspecified consecutive/random sample selection and unspecified patient exclusions. The majority of studies (27 of 31) did not report blinding regarding CSF a-syn quantification, whereas 8 studies did not control for CSF blood contamination. Despite these shortcomings, the risk of bias was considered low in all studies, since a-syn measurement cannot be manipulated, whereas only the studies with no control for CSF blood contamination were considered to have suboptimal applicability. The risk of bias for reference standard and flow/timing was generally low (Figure 2, Supplementary Table S1).

### 3.4. Results of Meta-Analysis

**Figure 3:**
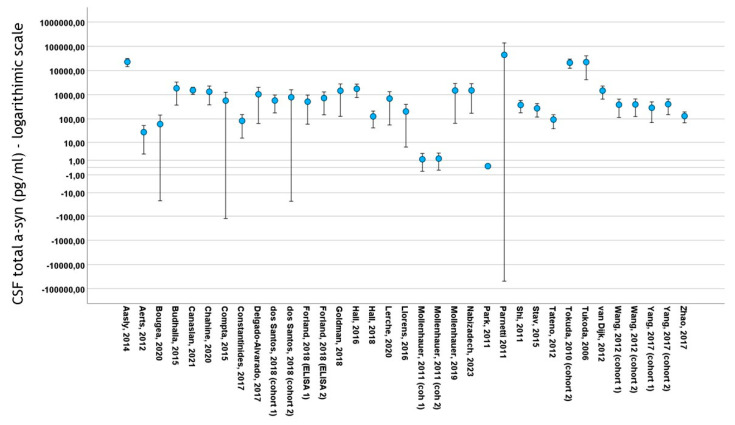
Error bars of mean ± 2 standard deviation values of total CSF a-syn (logarithmic scale) among studies included in meta-analysis. For citations of studies please refer to Table 1.

Thirty studies included data on total CSF a-syn in PD cohorts. A total of 4113 subjects (2475 PD patients and 1638 control subjects) were included in these studies. PD cohort samples ranged from 11 to 413 patients and control cohorts from 10 to 187 subjects. The mean total CSF a-syn ranged from 0.140 pg/mL to 44,400 pg/mL in PD cohorts and from 0.13 pg/mL to 69,800 pg/mL in control subjects (Figure 3). The mean age in PD cohorts varied from 54 to 73 years, mean disease duration from 10.4 to 146 months and male-to-female ratio from 0.92 to 3.65. The mean age in control cohorts varied from 47.4 to 73 years and the male-to-female ratio from 0.47 to 2.83

Twenty-one of the thirty-five cohorts (included in the thirty relevant studies) with CSF total a-syn data reported significantly lower levels of total a-syn in PD cohorts compared to control subjects. Thirteen studies did not report significant differences between PD and control groups, whereas a single study reported significantly increased total a-syn in the PD group compared to the control group. Cohen’s *d* ranged from −1.48 to 1.30. The overall Cohen’s *d* for total a-syn was −0.46 (SE = 0.08; *p* < 0.001).

A single study with two cohorts (with 209 PD patients and 204 control subjects) included data on phosphorylated a-syn. In both cohorts, PD patients exhibited significantly increased CSF phosphorylated a-syn levels compared to control subjects (overall Cohen’s *d* = 0.37; range 0.25–0.51; SE = 0.13; *p* = 0.01).

**Figure 4:**
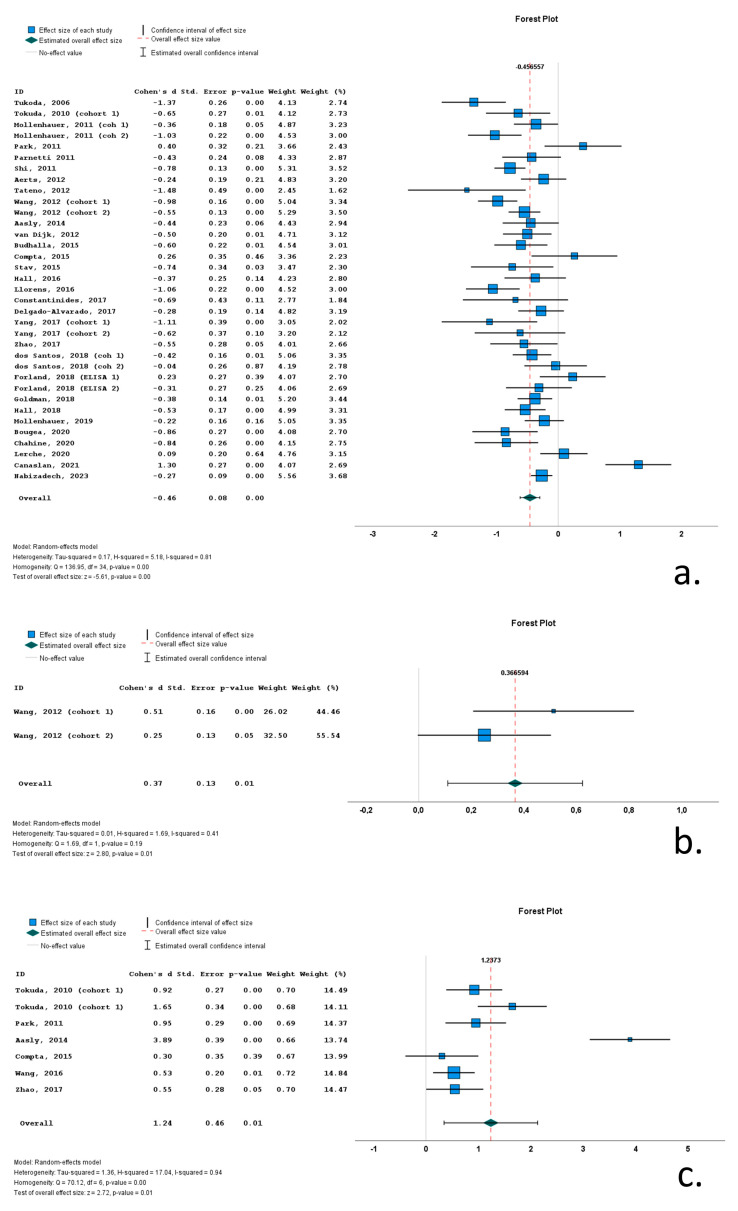
Forest plots of effect size (as measured by Cohen’s *d*) of studies on PD patients compared to control subjects for (**a**) CSF total a-syn; (**b**) CSF phosphorylated a-syn and (**c**) CSF oligomeric a-syn. For citations of studies please refer to Table 1.

Seven cohorts (in six studies) contained data on CSF oligomeric a-syn. These cohorts included 307 PD patients and 193 control subjects. The CSF oligomeric a-syn levels ranged from 0.33 pg/mL to 3.36 × 10^7^ pg/mL in PD cohorts. Six of the seven studies reported significantly increased CSF oligomeric a-syn levels in PD patients compared to control subjects. Cohen’s *d* ranged from 0.30 to 3.89. Overall Cohen’s *d* for total a-syn was 1.24 (SE = 0.46; *p* = 0.01) (Figure 4).

### 3.5. Heterogeneity

The *Q* statistic was used to qualitatively assess the presence or absence of heterogeneity, and the I^2^ statistic was applied to quantify between-study heterogeneity.

CSF total a-syn (*Q* statistic *p*-value < 0.001; *I*^2^ = 0.81) and oligomeric a-syn (*Q* statistic *p*-value < 0.001; *I*^2^ = 0.94) exhibited high heterogeneity. Phosphorylated a-syn did non exhibit heterogeneity (*Q* statistic *p*-value = 0.19; *I*^2^ = 0.41); however, only two cohorts included data on this metric.

### 3.6. Subgroup Analyses

In an effort to account for the high between-study heterogeneity in CSF total a-syn, subgroup analyses were conducted to explore the effect of categorical variables (possible moderators) on between-study variability.

**Figure 5:**
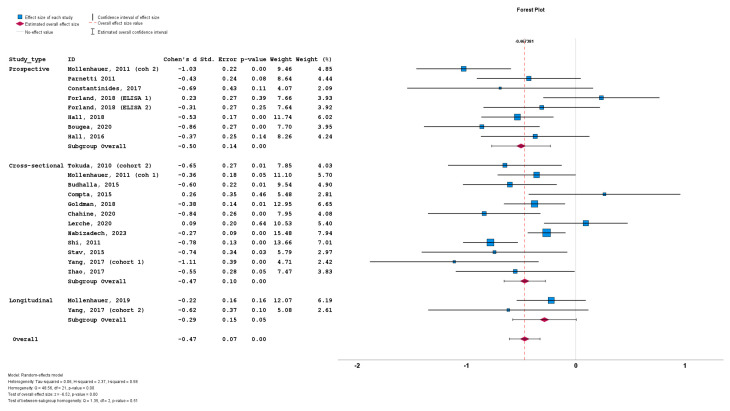
Forest plots of effect size (as measured by Cohen’s *d*) of studies on PD patients compared to control subjects for CSF total a-syn stratified by study design (i.e., prospective vs. cross-sectional vs. longitudinal). For citations of studies please refer to Table 1.

We initially stratified the studies based on study design, with no significant differences among the prospective cohorts (n = 8; subgroup Cohen’s *d* = −0.50; SE = 0.14; *p* < 0.001), cross-sectional cohorts (n = 12; subgroup Cohen’s *d* = −0.47; SE = 0.10; *p* < 0.001) and longitudinal cohorts (n = 2; subgroup Cohen’s *d* = −0.29; SE = 0.15; *p* = 0.05). No statistically significant subgroup difference was detected (*Q* = 1.35; *p* = 0.51) (Figure 5).

**Figure 6:**
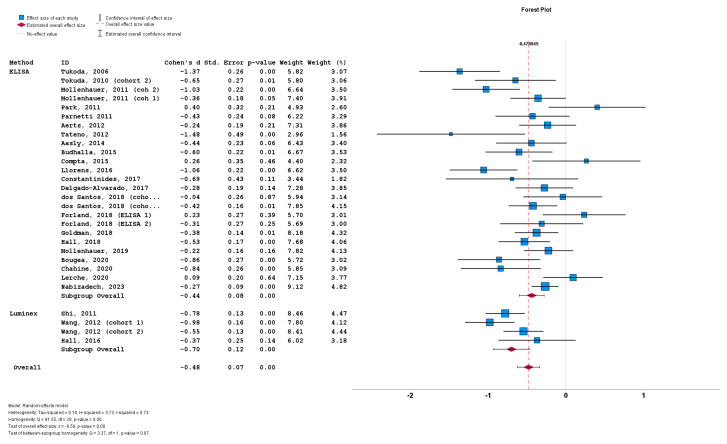
Forest plots of effect size (as measured by Cohen’s *d*) of studies on PD patients compared to control subjects for CSF total a-syn stratified by a-syn measurement method (i.e., ELISA vs. Luminex). For citations of studies please refer to Table 1.

The stratification of studies based on the a-syn measurement method did not result in significant differences between cohorts implementing ELISA (n = 26; subgroup Cohen’s *d* = −0.44; SE = 0.08; *p* < 0.001), and cohorts implementing Luminex (n = 4; subgroup Cohen’s *d* = −0.70; SE = 0.12; *p* < 0.001). No statistically significant subgroup difference was detected (*Q* = 3.37; *p* = 0.07) (Figure 6).

**Figure 7:**
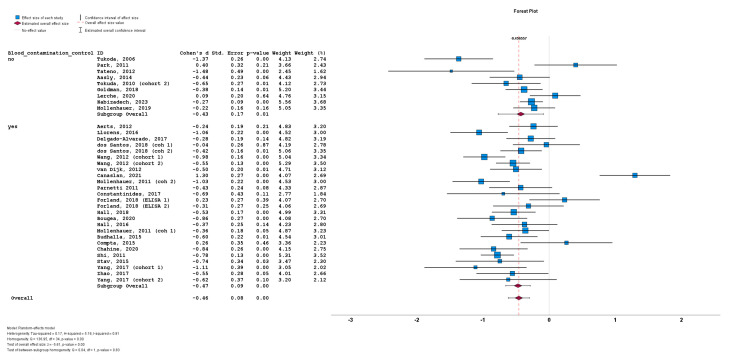
Forest plots of effect size (as measured by Cohen’s *d*) of studies on PD patients compared to control subjects for CSF total a-syn stratified by presence or absence of CSF blood contamination control. For citations of studies please refer to Table 1.

The stratification of studies based on the presence or absence of CSF blood contamination control for a-syn measurement also did not result in significant differences between cohorts implementing CSF blood contamination control (n = 26; subgroup Cohen’s *d* = −0.47; SE = 0.08; *p* < 0.001) or cohorts not implementing CSF blood contamination control (n = 9; subgroup Cohen’s *d* = −0.43; SE = 0.17; *p* = 0.01). No statistically significant subgroup difference was detected (*Q* = 0.04; *p* = 0.83) (Figure 7).

**Figure 8:**
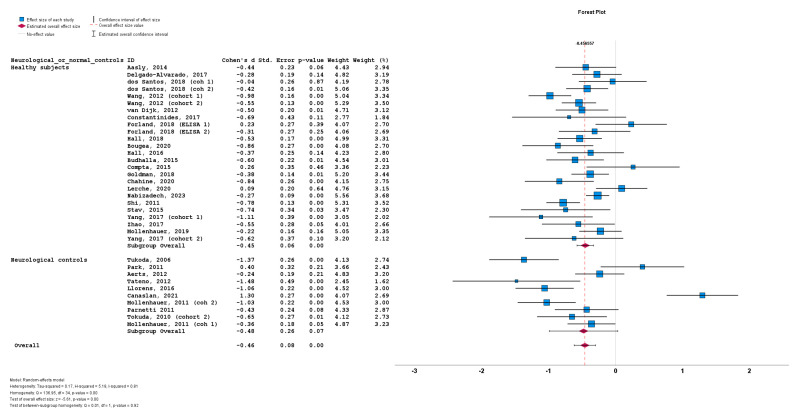
Forest plots of effect size (as measured by Cohen’s *d*) of studies on PD patients compared to control subjects for CSF total a-syn stratified by use of healthy subjects vs. neurological controls. For citations of studies please refer to Table 1.

Lastly, the stratification of studies based on the use of healthy subjects vs. neurological controls for a-syn measurement also did not result in significant differences between cohorts using healthy subjects (n = 25; subgroup Cohen’s *d* = −0.45; SE = 0.06; *p* < 0.001) or cohorts using neurological controls (n = 10; subgroup Cohen’s *d* = −0.48; SE = 0.26; *p* = 0.07). No statistically significant subgroup difference was detected (*Q* = 0.01; *p* = 0.92) (Figure 8).

### 3.7. Meta-Regression Analysis

Univariate random-effects meta-regression models were applied to explore the source of between-study heterogeneity. The continuous variables (possible moderators) of the PD cohorts included in these analyses were as follows: (a) male/female ratio; (b) mean age; (c) mean disease duration; (d) mean UPRDS III score; (e) mean H & Y score. The continuous variables of the control cohorts included the male/female ratio and mean age. Bubble plots were produced to illustrate the association between moderators and effect size.

**Figure 9:**
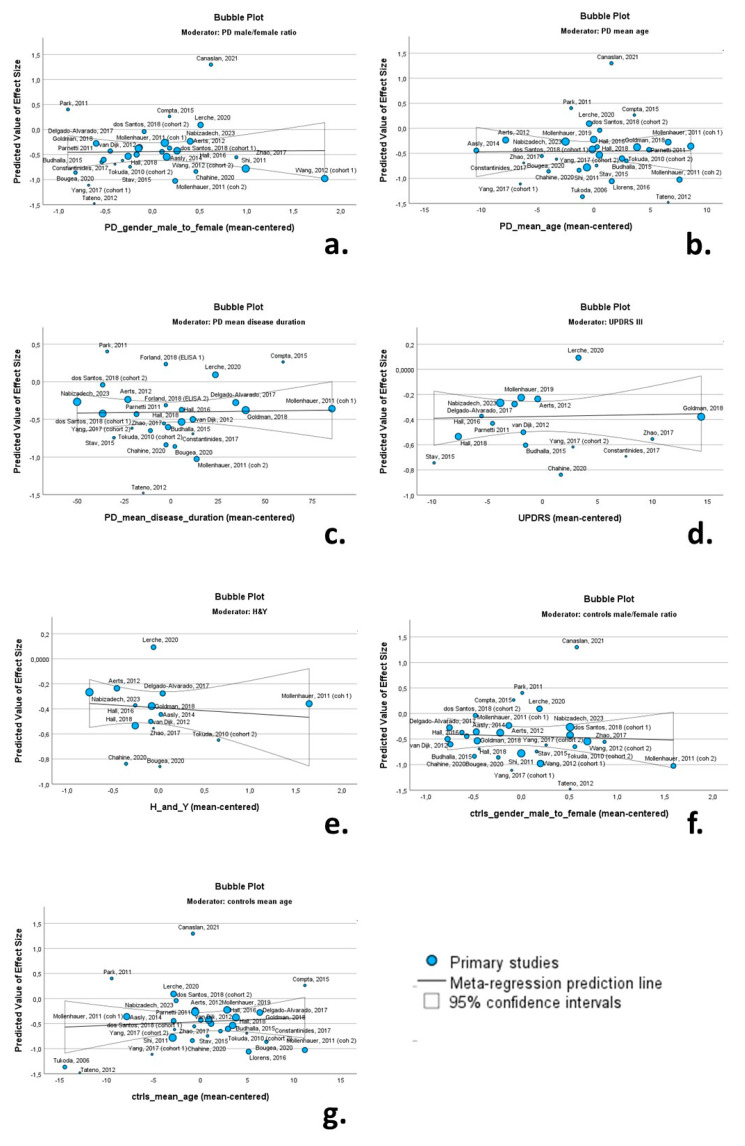
Bubble plots of associations between total a-syn (effect size expressed as Cohen’s *d*) and PD male/female ratio (**a**); PD mean age (**b**); PD mean disease duration (**c**); UPDRS II (**d**); H & Y (**e**); control group male/female ratio (**f**); and control group mean age (**g**). For citations of studies please refer to Table 1.

None of the continuous variables had a significant moderating effect on CSF total a-syn levels. More specifically the male/female ratio (*β* = 0.013; *p* = 0.931), mean age of PD cohorts (*β* = 0.001; *p* = 0.951), mean disease duration (*β* = 0.001; *p* = 0.892), UPDRS III score (*β* = 0.001; *p* = 0.867) and H & Y score (*β* = −0.046; *p* = 0.647) were not associated with Cohen’s *d* regarding total CSF a-syn. Likewise, the male/female ratio (*β* = −0.048; *p* = 0.762) and mean age of control cohorts (*β* = 0.007; *p* = 0.656) were not associated with Cohen’s *d* (Table 2). Relevant bubble plots are provided in Figure 9.

### 3.8. Publication Bias

**Figure 10:**
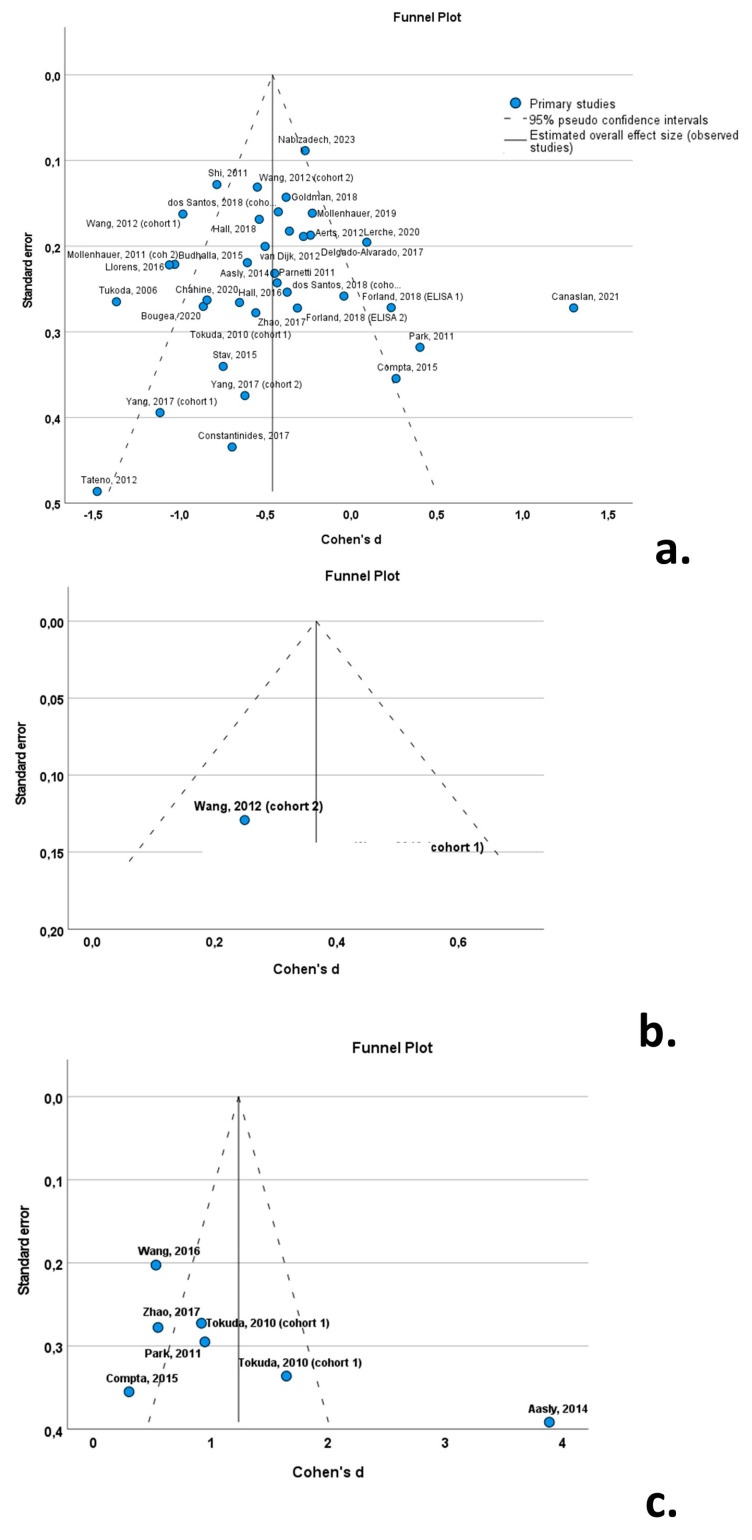
Forest plots of effect size (as measured by Cohen’s *d*) of studies on PD patients compared to control subjects for (**a**) CSF total a-syn; (**b**) CSF phosphorylated a-syn; and (**c**) CSF oligomeric a-syn. For citations of studies please refer to Table 1.

The funnel plots indicated a degree of asymmetry in PD patients for oligomeric a-syn, indicative of publication bias. However, the results of the Egger’ regression-based test did not support the presence of publication bias for CSF total a-syn (*β_0_* = −0.339; −0.847—0.169; *p*-value = 0.184) or oligomeric a-syn (*β_0_* = −2.552; −7.735—2.631; *p*-value = 0.261) (Figure 10).

## 4. Discussion

Parkinson’s disease is the most common neurodegenerative movement disorder [1]. In its typical manifestation, it is characterized by slowly progressive, asymmetric, 4–6 Hz upper limb rest tremor, bradykinesia and rigidity. However, a subset of PD patients exhibit a predominantly rigid-akinetic syndrome, without tremor [2]. Moreover, progressive supranuclear palsy—Parkinsonism (PSP-P) and multiple system atrophy—parkinsonian type (MSA-P) frequently present with phenotypes identical to PD, particularly in the early disease stages, rendering the differential diagnosis between PD and atypical parkinsonian syndromes difficult [43,44]. Thus, the diagnostic accuracy for PD, based on established clinical diagnostic criteria, is suboptimal [45]. To this end, the introduction of a biomarker with molecular specificity to assist in a timely and accurate clinical diagnosis is pivotal, considering the low specificity of clinical diagnosis of PD based on diagnostic criteria, even among movement disorder experts [46].

In addition to their clinical significance, biomarkers are paramount in advancing our understanding of the underlying pathophysiological mechanisms driving neurodegeneration at the preclinical or oligosymptomatic phases in PD. This has already been evidenced in subjects with REM sleep behavior disorder [47], as well as in asymptomatic carriers of pathogenic PD mutations, such as mutations in the a-syn gene (SCNA) [48]. However, to date, a biomarker with molecular specificity for PD is lacking. To this end, multiple studies have focused on different forms of a-syn as candidate biomarkers of PD. These approaches have included total, phosphorylated and oligomeric CSF a-syn, aggregated and exosome-associated a-syn, and plasma and red blood cell a-syn, among others [29,49,50,51]. This review aimed to present data regarding CSF total, phosphorylated and oligomeric a-syn in PD cohorts.

An initial conclusion of this systematic review is that most studies to date have focused on total a-syn (thirty studies), followed by oligomeric a-syn (six studies). A single study on phosphorylated a-syn fulfilled the inclusion criteria of this review.

A second conclusion was that the studies included were heterogeneous regarding their study design (i.e., prospective vs. retrospective vs. cross-sectional vs. longitudinal), the assay used for CSF a-syn measurement (ELISA vs. Luminex vs. other), the presence or absence of a control for CSF blood contamination before a-syn quantification, and the use of healthy subjects or patients with non-degenerative neurological disorders as control subjects. The heterogeneity among studies was confirmed statistically, using the *Q* statistic and *I*^2^ value, both for total and oligomeric a-syn.

Subgroup analyses were performed using these four variables (study design, method of a-syn measurement, control for CSF blood contamination, type of control group) to investigate the effect of these possible moderators on the high between-study heterogeneity. However, none of these analyses indicated a significant effect of these methodological factors on CSF total a-syn levels, indicating that methodological variability is not a significant cause of between-study heterogeneity.

In an effort to further investigate the causes of heterogeneity, meta-regression was performed using multiple continuous clinical and demographic variables as possible moderators, including the PD cohorts’ mean age, disease duration, the male/female ratio and the UPDRS III and H & Y scales, as well as control cohorts’ mean age and male/female ratio. None of these moderators had a significant association with total CSF a-syn, indicating that clinical variability was not a significant cause of between-study heterogeneity.

Importantly, the mean values of total CSF a-syn varied considerably among studies (range: 0.140 pg/mL to 44,400 pg/mL). The same pattern was evident for oligomeric CSF a-syn (range: 0.33 pg/mL to 3.36 × 10^7^ pg/mL). Taking into account the minimal effect of both methodological and clinical variability on between-study heterogeneity, the great aforementioned variability in CSF a-syn mean levels across studies could be attributed to (a) pre-analytical factors (e.g., types of tubes, CSF spinning conditions, freeze/thaw cycles, time of lumbar puncture, CSF volume withdrawal, type of needle used for lumbar puncture, aliquot tube volume and filling, freezing temperature, etc.) and (b) analytical factors (i.e., differences in capture and detection antibodies). This is further supported by the significant variability in CSF a-syn levels determined using ELISA, indicating that the lack of standardization in these (pre)analytical factors could be the main cause of heterogeneity.

Total CSF a-syn levels were decreased in PD patients compared to control subjects (overall Cohen’s *d*: −0.46; SE = 0.08; *p* < 0.001), indicating a small to medium effect size of total a-syn. Moreover, only 21 of the 35 included cohorts reported statistically lower levels of total a-syn in PD compared to controls, with 13 studies reporting no difference and a single study reporting an increase in total a-syn in PD compared to controls. Additionally, the specificity and sensitivity of total a-syn for the differentiation of PD from control subjects was suboptimal in all included studies. These data indicate that PD patients exhibit a decrease in CSF total a-syn compared to healthy subjects; however, the differences in total a-syn levels between the two groups are marginal due to significant overlap, and thus, total a-syn cannot be utilized as a biomarker for PD.

Contrary to total a-syn, oligomeric a-syn is increased in PD cohorts compared to control groups. Six of the seven included studies reported a statistically significant increase in oligomeric a-syn in PD (overall Cohen’s *d*: 1.24; SE = 0.46; *p* = 0.01). The effect size was very large. However, these results on oligomeric a-syn are based on considerably smaller cohorts (307 PD patients and 193 control subjects in total) compared to total a-syn (2475 PD patients and 1638 control subjects). Safe conclusions regarding phosphorylated a-syn in PD cannot be reached, due to the scarcity of studies.

There are certain limitations to this systematic review and meta-analysis. Initially, due to the statistical design of our meta-analysis and the use of Cohen’s *d* as a measure of standardized mean difference, only studies with available mean values and standard deviations regarding CSF a-syn levels were included. Thus, several studies without availability of these data were excluded. We made every effort to extract relevant data form scatterplots where available and seek raw data from the authors to compensate for this limitation. An additional limitation was the absence of a neuropathological diagnosis of PD. All included studies relied on clinical diagnostic criteria to establish a PD diagnosis. With a lack of neuropathological confirmation, the misdiagnosis of patients is possible. However, most studies reported the inclusion of well-characterized cohorts with adequate follow-up. For the purposes of this review, we opted to exclude genetic PD cohorts and only included cohorts of patients with sporadic PD, due to possible neurochemical differences between patients with sporadic and genetic PD. Lastly, we did not include data regarding seeding assays such as PMCA or RTQuIC. These assays are very promising and may represent a breakthrough in the effort to establish a biomarker for synucleinopathies. However, they provide qualitative results but do not produce quantifiable results. For this reason, they could not be incorporated into the statistical analysis of the present study.

This meta-analysis reaffirms that PD patients exhibit a decrease in total CSF a-syn and an increase in phosphorylated and oligomeric a-syn compared to healthy subjects or neurological control groups. However, the significant overlap in CSF a-syn levels between PD and control groups results in suboptimal sensitivity and specificity and indicates that a-syn, as measured by the established assays, cannot be used clinically as a biomarker for PD. The standardization of pre-analytical (e.g., CSF blood contamination) and analytical variables (e.g., assay used) in a-syn measurement may further improve its diagnostic accuracy for PD [52]. In addition to a-syn, the implementation of biomarkers for other proteinopathies (e.g., Alzheimer’s disease) and the use of biomarker classification systems, such as AT(N) may assist in the identification of patients with dual pathologies and further enhance the in vivo diagnosis of PD [5,53,54]. The development of novel biomarkers or novel assays is needed to advance the research field of PD biomarkers. To this end, seeding assays for a-syn quantification have provided promising results regarding the in vivo diagnosis of PD and may represent a breakthrough in the field [55,56].

## Figures and Tables

**Table 1:** Characteristics of studies included in meta-analysis. Period recruit.: period of patient recruitment; CSF blood contam.: Presence or absence of control for CSF blood contamination prior to a-syn quantification; C: cross-sectional; P: prospective; L: longitudinal; R: retrospective; PD: Parkinson’s disease; U: unspecified; t-a-syn: total a-syn; ph-a-syn: phosphorylated a-syn; o-a-syn: oligomeric a-syn; AUC: area under the curve.

Study Num	Study	Study Design	Period Recruit.	Study Origin	Method a-syn	CSF Blood Contam.	Available Data	Main Findings
1	Tukoda, 2006 [12]	U		Japan	ELISA	no	total	t-a-syn PD < ctrl (AUC: 0.815)
2	Tokuda, 2010 [13]	C		Japan	ELISA	no	total, oligomeric	o-a-syn PD > ctlrs (sens: 75%; spec: 87.5%) o-a-syn/t-a-syn PD > ctrls (sens: 89.3%; spec: 90.6%)
3	Mollenhauer, 2011 [14]	C and P 2 cohorts	2003–2006	Two centers (Germany; USA)	ELISA	yes	total	t-a-syn PD < ctrl (sens: 70.7%; spec: 52.8%)
4	Park, 2011 [15]	U		Korea	ELISA	no	total, oligomeric	o-a-syn PD > controls
5	Parnetti, 2011 [16]	P	2005–2009	Italy	ELISA	yes	total	t-a-syn PD < controls (sens: 94%; spec: 25%)
6	Shi, 2011 [17]	C		Multicenter	Luminex	yes	total	t-a-syn PD < ctrls (sens: 92%; spec: 38%)
7	Aerts, 2012 [18]	U	2003–2006	Netherlands	ELISA	yes	total	t-a-syn PD ≈ ctrls
8	Tateno, 2012 [19]	U		Japan	ELISA	no	total	t-a-syn PD ≈ ctrls
9	Wang, 2012 [38]	U		Multicenter	Luminex	yes	total, phosphorylated	t-a-syn PD < ctrls (sens: 82%; spec: 63%) ph-a-syn > ctrls (sens: 86%; spec: 30%)
10	Aasly, 2014 [32]	U		Norway	ELISA	no	total, oligomeric	o-a-syn > controls (sens: 65%; spec: 83%)
11	van Dijk, 2014 [20]	U	2008–2011	Norway	TR-FRET immunoassay	yes	total	t-a-syn PD < ctrls (sens: 56%; spec: 74%)
12	Budhalla, 2015 [21]	C	2011–2014	USA	ELISA	yes	total	t-a-syn PD < ctrls
13	Compta, 2015 [22]	C		Spain	ELISA	yes	total, oligomeric	t-a-syn PD ≈ ctrls
14	Stav, 2015 [23]	C	2011–2014	Norway	electrochemiluminescence	yes	total	t-a-syn PD < ctrls
15	Hall, 2016 [24]	P		Sweden	ELISA	yes	total	No data
16	Llorens, 2016 [33]	U		Germany	ELISA	yes	total	t-a-syn PD < ctrls (sens: 67%; spec: 98%)
17	Wang, 2016 [34]	R	2013–2014	China	ELISA	no	oligomeric	o-a-syn PD > ctrls
18	Constantinides, 2017 [25]	P		Greece	ELISA	yes	total	t-a-syn PD ≈ ctrls
19	Delgado-Alvarado, 2017 [35]	U		Spain	ELISA	yes	total	t-a-syn PD < ctrlsAUC: 0.685
20	Yang, 2017 [39]	C and L2 cohorts		Multicenter	Luminex	yes	total	No data
21	Zhao, 2017 [26]	C		China	AlphaLISA	yes	total, oligomeric	t-a-syn PD ≈ ctrlso-a-syn PD ≈ ctrls
22	dos Santos, 2018 [27]	U		Germany	ELISA	yes	total	t-a-syn PD < ctrls (cohort 1), t-a-syn PD ≈ ctrls (cohort 2)
23	Forland, 2018 [28]	P		Norway	ELISA	yes	total	t-a-syn PD ≈ ctrls
24	Goldman, 2018 [29]	C		Multicenter	ELISA	no	total	t-a-syn PD < ctrls
25	Hall, 2018 [40]	P		Sweden	ELISA	yes	total	t-a-syn PD ≈ ctrls
26	Mollenhauer, 2019 [41]	L	2009–2012	Germany	ELISA	no	total	t-a-syn PD ≈ ctrls
27	Bougea, 2020 [36]	P	2013–2016	Greece	ELISA	yes	total	t-a-syn PD < ctrls (sens: 78%; spec: 57%)
28	Chahine, 2020 [37]	C	2015–2017	Multicenter	ELISA	yes	total	t-a-syn PD < ctrls (sens: 87%; spec: 63.2%)
29	Lerche, 2020 [30]	C	2005–2008	Germany	ELISA	no	total	t-a-syn PD ≈ ctrls
30	Canaslan, 2021 [42]	U		Multicentre	SIMOA	yes	total	t-a-syn PD ≈ ctrls
31	Nabizadeh, 2023 [31]	C		Multicentre	ELISA	no	total	t-a-syn PD < ctrls

**Table 2:** Meta-regression analysis exploring effect of PD and control cohort characteristics on CSF total a-syn levels.

	n	*β*	SE	*p* Value	95% Confidence Interval		*Q*	*I* ^2^
					Lower Bound	Upper Bound		
PD characteristics								
Male/female ratio	30	0.013	0.145	0.931	−0.285	0.310	105.4	80.0
Mean age	30	0.001	0.021	0.951	−0.042	0.045	118.9	82.2
Mean disease duration	26	0.000	0.002	0.892	−0.004	0.004	50.2	52.6
UPDRS III	16	0.001	0.009	0.867	−0.017	0.020	16.8	18.8
H & Y	14	−0.046	0.098	0.647	−0.259	0.167	16.6	22.6
Control group characteristics								
Male/female ratio	29	−0.048	0.158	0.762	−0.371	0.275	109.1	80.8
Mean age	31	0.007	0.016	0.656	−0.026	0.041	119.4	81.6

## Supplementary Materials

The following supporting information can be downloaded at: https://www.mdpi.com/article/10.3390/biomedicines12102266/s1, Supplementary Table S1: Quality assessment of studies.
