# Supplementary material for: Cerebrospinal Fluid Total, Phosphorylated and Oligomeric A-Synuclein in Parkinson’s Disease: A Systematic Review, Meta-Analysis and Meta-Regression Study
Source: Biomedicines. 2024 Oct 5;12(10):2266. doi: 10.3390/biomedicines12102266 (PMC11504870; doi:10.3390/biomedicines12102266)
Supplement: Supplementary file 1 [file biomedicines-12-02266-s001.zip › Supplementary Table 1.pdf]

		Patient Selection				Index Test				Reference Standard			Flow and Timing			
		Consecutive / random sample	Innapropriate exclusions?	ROB	App	Test assessed blindly	CSF blood contamination control	ROB	App	Is the RS likely to correctly classify participants	ROB	App	All patients received RS	All patients received same RS	All patient included?	ROB
1	Tokuda, 2016	?	☺	?	☺	?	☹	☺	☹	☺	☺	☺	☺	☺	☺	☺
2	Tokuda 2010	?	☺	?	☺	?	☹	☺	☹	☺	☺	☺	☺	☺	☺	☺
3	Mollenhauer, 2011	☺	☺	☺	☺	?	☺	☺	☺	☺	☺	☺	☺	☺	☺	☺
4	Park, 2011	☺	☺	☺	☺	?	☺	☺	☺	☺	☺	☺	☺	☺	☺	☺
5	Parnetti, 2011	☺	☺	☺	☺	?	☺	☺	☺	☺	☺	☺	☺	☺	☺	☺
6	Shi, 2011	?	☹	☹	☹	?	☺	☺	☺	☺	☺	☺	☺	☺	☺	☺
7	Aerts, 2012	☺	☺	☺	☺	☺	☺	☺	☺	☺	☺	☺	☺	☺	☺	☺
8	Tateno, 2012	☺	☺	☺	☺	?	☺	☺	☺	☺	☺	☺	☺	☺	☺	☺
9	Wang, 2012	?	☺	?	☺	?	☺	☺	☺	☺	☺	☺	☺	☺	☺	☺
10	Aasly, 2014	?	☺	?	☺	?	☹	☺	☹	☺	☺	☺	☺	☺	☺	☺
11	van Dijkk, 2014	?	☺	?	☺	?	☺	☺	☺	☺	☺	☺	☺	☺	☺	☺
12	Buddhala, 2015	?	☺	?	☺	☺	☺	☺	☺	☺	☺	☺	☺	☺	☺	☺
13	Compta, 2015	?	☺	?	☺	?	☺	☺	☺	☺	☺	☺	☺	☺	☺	☺
14	Stav, 2015	☺	☺	☺	☺	?	☺	☺	☺	☺	☺	☺	☺	☺	☺	☺
15	Hall, 2016	?	☺	?	☺	☺	☺	☺	☺	☺	☺	☺	☺	☺	☺	☺
16	Llorens, 2016	☺	☺	☺	☺	?	☺	☺	☺	☺	☺	☺	☺	☺	☺	☺

17	**Wang, 2016**	?	☺	?	☺	?	☺	☺	☺	☺	☺	☺	☺	☺	☺	☺
18	**Constantinides, 2017**	?	☺	?	☺	?	☺	☺	☺	☺	☺	☺	☺	☺	☺	☺
19	**Delgado-Alvarado, 2017**	?	☺	?	☺	?	☺	☺	☺	☺	☺	☺	☺	☺	☹	☹
20	**Yang, 2017**	?	☺	?	☺	?	☺	☺	☺	☺	☺	☺	☺	☺	☺	☺
21	**Zhao, 2017**	?	☺	?	☺	?	☹	☺	☹	☺	☺	☺	☺	☺	?	☺
22	**Dos Santos, 2018**	?	☺	?	☺	?	☺	☺	☺	☺	☺	☺	☺	☺	☺	☺
23	**Forland, 2018**	?	☺	?	☺	?	☺	☺	☺	☺	☺	☺	☺	☺	?	☺
24	**Goldman, 2018**	?	☺	?	☺	?	☹	☺	☹	☺	☺	☺	☺	☺	?	☺
25	**Hall, 2018**	?	☺	?	☺	☺	☺	☺	☺	☺	☺	☺	☺	☺	☺	☺
26	**Mollenhauer, 2019**	?	☺	?	☺	?	☹	☺	☹	☺	☺	☺	☺	☺	☹	☹
27	**Bougea, 2020**	☺	☺	☺	☺	?	☺	☺	☺	☺	☺	☺	☺	☺	☺	☺
28	**Chahine, 2020**	?	☺	?	☺	?	☺	☺	☺	☺	☺	☺	☺	☺	☺	☺
29	**Lerche, 2020**	?	☺	?	☺	?	☹	☺	☹	☺	☺	☺	☺	☺	☺	☺
30	**Canaslan, 2021**	?	☺	?	☺	?	☺	☺	☺	☺	☺	☺	☺	☺	☺	☺
31	**Nabizadeh, 2023**	?	☺	?	☺	?	☹	☺	☹	☺	☺	☺	☺	☺	☺	☺

Supplementary Table 1. Quality assessment of studies, based on the QUADAS-2 test; ROB: Risk of Bias; App: Concerns regarding applicability; ☺: low; ☹: high; ?: unclear
